# High-throughput sequencing and analysis of the gill tissue transcriptome from the deep-sea hydrothermal vent mussel *Bathymodiolus azoricus*

**DOI:** 10.1186/1471-2164-11-559

**Published:** 2010-10-11

**Authors:** Raul Bettencourt, Miguel Pinheiro, Conceição Egas, Paula Gomes, Mafalda Afonso, Timothy Shank, Ricardo Serrão Santos

**Affiliations:** 1 University of the Azores, Department of Oceanography and Fisheries, 9901-861 Horta, Portugal; 2 IMAR Institute of Marine Research, 9901-861 Horta, Portugal; 3 Bioinformatics Unit, Biocant, 3060-197 Cantanhede, Portugal; 4 Advanced Services Unit, Biocant, 3060-197 Cantanhede, Portugal; 5 Center for Neurosciences and Cellular Biology, University of Coimbra, 3004-517 Coimbra, Portugal; 6 Woods Hole Oceanographic Institution, Woods Hole, MA 02543, USA

## Abstract

**Background:**

*Bathymodiolus azoricus *is a deep-sea hydrothermal vent mussel found in association with large faunal communities living in chemosynthetic environments at the bottom of the sea floor near the Azores Islands. Investigation of the exceptional physiological reactions that vent mussels have adopted in their habitat, including responses to environmental microbes, remains a difficult challenge for deep-sea biologists. In an attempt to reveal genes potentially involved in the deep-sea mussel innate immunity we carried out a high-throughput sequence analysis of freshly collected *B. azoricus *transcriptome using gills tissues as the primary source of immune transcripts given its strategic role in filtering the surrounding waterborne potentially infectious microorganisms. Additionally, a substantial EST data set was produced and from which a comprehensive collection of genes coding for putative proteins was organized in a dedicated database, "DeepSeaVent" the first deep-sea vent animal transcriptome database based on the 454 pyrosequencing technology.

**Results:**

A normalized cDNA library from gills tissue was sequenced in a full 454 GS-FLX run, producing 778,996 sequencing reads. Assembly of the high quality reads resulted in 75,407 contigs of which 3,071 were singletons. A total of 39,425 transcripts were conceptually translated into amino-sequences of which 22,023 matched known proteins in the NCBI non-redundant protein database, 15,839 revealed conserved protein domains through InterPro functional classification and 9,584 were assigned with Gene Ontology terms. Queries conducted within the database enabled the identification of genes putatively involved in immune and inflammatory reactions which had not been previously evidenced in the vent mussel. Their physical counterpart was confirmed by semi-quantitative quantitative Reverse-Transcription-Polymerase Chain Reactions (RT-PCR) and their RNA transcription level by quantitative PCR (qPCR) experiments.

**Conclusions:**

We have established the first tissue transcriptional analysis of a deep-sea hydrothermal vent animal and generated a searchable catalog of genes that provides a direct method of identifying and retrieving vast numbers of novel coding sequences which can be applied in gene expression profiling experiments from a non-conventional model organism. This provides the most comprehensive sequence resource for identifying novel genes currently available for a deep-sea vent organism, in particular, genes putatively involved in immune and inflammatory reactions in vent mussels.

The characterization of the *B. azoricus *transcriptome will facilitate research into biological processes underlying physiological adaptations to hydrothermal vent environments and will provide a basis for expanding our understanding of genes putatively involved in adaptations processes during post-capture long term acclimatization experiments, at "sea-level" conditions, using *B. azoricus *as a model organism.

## Background

Deep-sea hydrothermal vent ecosystems are driven by unique physical, geochemical and biological processes with specialized energy sources at the origin of the trophic web. Since the discovery of hydrothermal vents and their associated fauna in the Galapagos Rift, evidence of the establishment of dense faunal communities based on chemosynthesis have mounted over the past decades, and generally in relation to areas where tectonic movements and deep ocean volcanism are active [1]. Hydrothermal vent ecosystems are characterized by the synthesis of organic matter by means of chemo-autotrophic bacteria using reduced elements extracted from the hydrothermal fluids as source of energy [2,3].

Mussels in the genus *Bathymodiolus *are biomass dominant at many known deep-sea hydrothermal vent and cold seep habitats. Survival in such extreme conditions requires unique anatomical and physiological adaptations. For example the development of specialized gill epithelial cells harboring methanotrophic and thiotrophic endosymbiont bacteria constitutes one the best recognized adaptation strategies to chemosynthetic environments [4]. Dual symbiosis thus provides a clear nutritional advantage to Bathymodiolid mussels, allowing them to obtain energy from both sulfide and methane at the vent sites [5-7]. Near the Mid-Atlantic Ridge, and in the vicinity of the Azores region, *Bathymodiolus azoricus *subsists at vent sites, amid unusual levels of heavy metals, pH, temperature, CO_2_, methane and sulfide, while coping successfully with environmental microbes [8].

Despite its prominence as a model to study physiological adaptation to extreme physical and chemical conditions [9], there is currently no large scale genome project for *Bathymodiolus *species. Gene expression profiles are limited to a few EST projects mainly originated from the Evolution and Genetics of Marine Populations team at the Biological Station of Roscoff, France. In a recent analysis, the screening of cDNA libraries from whole bodies of *B. azoricus *resulted in 362 contigs and 1,918 singletons. Many genes known to be involved in both metallic and oxidative stress responses were then identified [10]. However, these data remain private and there is currently no published reports based on those sequences. More recently, the effect of temperature on the vent mussel *B. thermophilus *was investigated by means of subtractive suppression hybridization experiments aimed at the identification of genes differentially expressed in response to different temperatures regimes [11].

Thus far, knowledge of deep-sea biology or of the molecules involved in the maintenance of homeostasis in hydrothermal vent animals has been limited in part by the lack of information about their genomes and systematic sequencing of expressed sequence tags to identify protein coding genes on a large scale. The deep-sea vent biological systems represent the opportunity to study and provide new insights into the basic physiological principles that govern the defense mechanisms in vent animals and to understand how they cope with microbial infections. The problem of microbial threat and the need for immunity exist in both deep sea and shallow water bivalves however differences in the genes of marine organisms living in so distinct habitats are likely to occur. In order to significantly increase the number of *B. azoricus *genes in the public database and to discover new deep-sea vent adaptation-related genes in *B. azoricus*, and particularly for immune-related genes we have conducted a high-throughput experimental approach using pyrosequencing, on the 454 GS FLX (Roche-454 Life Sciences) with Titanium chemistry, to sequence the transcriptome of *B. azoricus *gill tissues. In the absence of a reference genome, this sequence method, which has not yet been widely applied to hydrothermal vent animals, holds great potential for discovery of genes and genetic markers in unconventional model species through *de novo *transcriptome sequence assembly. The assembled and annotated sequences were produced and have been organized in a dedicated database, accessible through the website, http://transcriptomics.biocant.pt:8080/deepSeaVent providing an extensive catalog of genes expressed in gill tissues harboring immune cells, the hemocytes, of the deep-sea vent mussel *Bathymodiolus azoricus*.

## Results and Discussion

### Sequence analysis

A cDNA library was constructed from mRNA of fresh gill tissues from *Bathymodiolus azoricus *collected at deep-sea vents and sequenced in a single GS FLX Titanium plate. A total of 778,996 raw nucleotide reads were produced with an average length of 283 bp, corresponding to 223.7 Mb. After removal of the SMART adaptors, by a custom script, the sequences were assembled with the MIRA software. A total of 582,650 quality reads were assembled into 75,407 contigs of which 3,071 were singletons corresponding to a total of 38,8 Mb. The length of the consensus sequences varied from 40 bp to 3,400 bp, with an average length of 509 bp. A summary of data is indicated in Table 1.

**Table 1:** Summary of assembly and EST data

Number of Reads	582,650
Total Bases	181 Mb
Average read length after MIRA	312
Number of contigs	75,407
Average contig length	509
Range contig length	40-3,400
Number of singletons	3,071
Number of Contigs with 2 reads	29,206
Number of Contigs with > 2 reads	43,130
Contigs with BLASTx matches (E-value ≤ 10^-6^)	18,407
*Remaining contigs with additional matches (E-value ≤ 10^-2^)	3,616
Contigs determined by ESTscan	17,402
**Total number of transcripts	39,425
**Total number of putatively translated amino-acids sequences	42,073

*contigs without BLASTx matches at an E-value cut-off of 10^-6 ^were queried again with BLASTx with an E-value cut-off of 10^-2^

** The difference between the number of transcripts and total number of amino-acid sequences is due to the possibility of a contig having more than one annotated protein hit.

**Figure 1:**
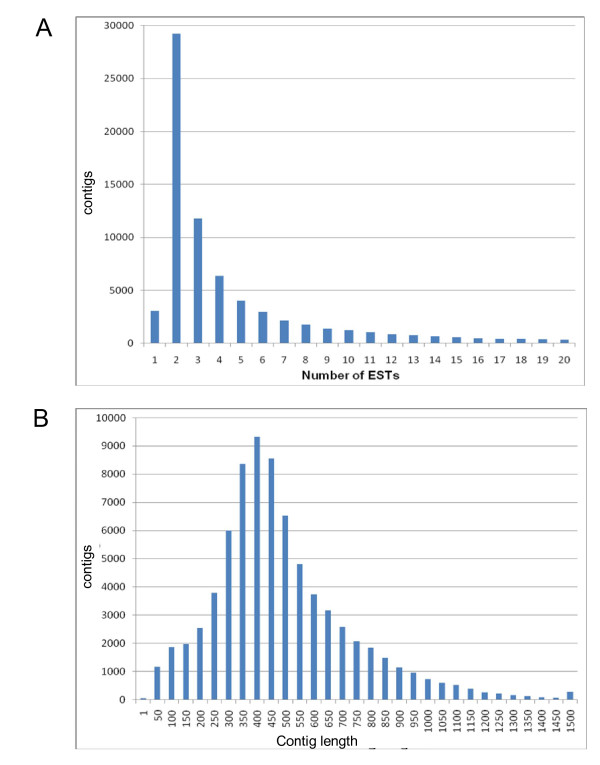
***B. azoricus *transcriptome assembly**. (A) Size distribution of 454 sequences after assembly and contig joining. (B) Distribution of number of read per contig in normalized library. The number of contigs presenting the indicated amount of reads is plotted as a histogram.

3,416 contigs were longer than 1 Kb, and 270 longer than 1.5 Kb. The distribution of contig length and EST assembly by contig are shown in Fig 1.

### Functional annotation

**Figure 2:**
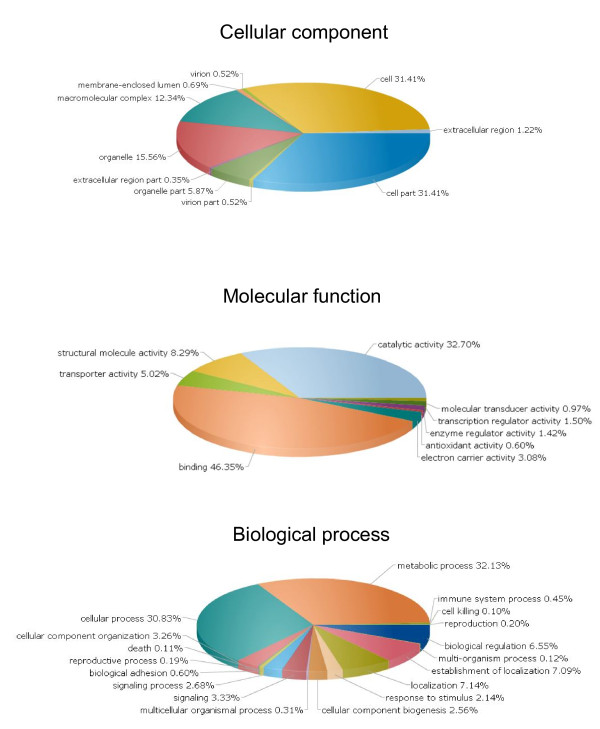
**Classification of the annotated amino-acid sequences**. Amino-acid sequences were grouped into different functional sub-categories within the Cellular Component, Molecular Function and Biological process Gene Ontology (GO) organizing principles.

The contigs were annotated taking into consideration the identity of the translation frame, the conserved protein domains and Gene Ontology (GO) terms. To obtain the translation frame, all contigs were queried against the NCBI protein database (nr) using BLASTx algorithm [12], resulting in 18,407 significant matches for an E-value ≤ 10^-6^. The process was repeated for contigs without hits, increasing the E-value cut-off to 10^-2^, and resulting in the identification of 3,616 additional contigs. The remaining contigs were processed through the ESTscan application [13], from which it was possible to identify the coding frame for 17,402 additional contigs. A total of 42,073 putative amino-acids sequences was obtained following this approach. The entire set of amino-acid sequences hits was queried against the InterPro database of protein families and functional domains http://www.ebi.ac.uk/InterPro[14,15], from which 15,839 were identified as bearing conserved protein domains. The same set of sequences was annotated with Gene Ontology (GO) terms resulting in 9,584 functional assignments according to the organizing principles of GO describing gene products and their properties. This classification scheme was useful to assign *Bathymodiolus *contigs to one of the major GO annotation categories, i.e., Biological Processes, Cellular Components and Molecular Functions in a species-independent manner [16] (Fig 2).

The largest proportion of GO assigned sequences fell into broad categories for all three major GO functional domains as presented in Fig 2. Within the Biological Process, 31% and 32% of assignments corresponded to "Cellular Process" (GO:0009987) and "Metabolic Process (GO:0008152) respectively, followed by the "Localization" (GO:0051179, 7%) and "Establishment of Localization" (GO:0051234, 7%) GO categories. Furthermore, the matches of molecular function terms were most prevalent within the "Binding" (GO:0005488, 46%) and "Catalytic Activity" (GO:0003824, 33%), followed by the categories "Structural Molecule Activity" (GO:0005198, 8%) and "Transporter Activity" (GO:0005215, 5%). Finally for the Cellular Component GO the most evident matches were within the "Cell Part" (GO:0044464, 31%) and "Cell" (GO:0005623, 31%) terms, followed by "Organelle" (GO:0043226, 16%) and "Macromolecular Complex" (GO:0032991, 12%). Together, these GO classes accounted for most of the assignable transcripts, and may represent a general gene expression profile signature for *B. azoricus *from the Lucky Strike hydrothermal vent field.

The contigs without any homology may correspond to one of the following categories: a) novel or diverged amino acid coding sequences that are specific to *Bathymodiolus *species, b) represent mostly 3' or 5' untranslated regions (UTRs) that would lack protein matches as they are non-coding or c) contain sequences to short to result in significant hits.

Despite that the Gene Ontology project is aimed at describing gene product characteristics and gene product annotation data, by ways of a direct acyclic graph (DAG) structure of controlled vocabularies, the process of assigning GO terms to gene products might prove difficult given the current GO structured vocabulary and the systematic relationship between the GO terms. This follows from the fact that GO describes how gene products behave in a cellular context and thus a gene product might be associated with or located in one or more cellular components; it is active in one or more biological processes, during which it performs one or more molecular functions.

### Immunity, stress and bacterial genes in *Bathymodiolus azoricus *gills

The innate immune system acts to protect the individual from invasive bacteria, viruses, and eukaryotic pathogens by detecting molecular signatures of infection and initiating effector responses. It probably originated early in animal phylogeny and is intimately related with wound healing and tissue maintenance functions among invertebrates [17]. This defense system is essential for the survival of invertebrates which do not possess immunoglobulins and rely on unique capabilities to detect and respond to microbe associated molecular patterns (MAMPs) such as lipopolysaccharides (LPS), lipoteichoic acids, lipoproteins, peptidoglycan (PGN) and (1→3) β-D-glucans [18] present on the cell surface of microorganisms. Upon recognition of the MAMPs, signal transduction molecules and transcription factors are set to mediate and control the expression of immune effector molecules, for example, the antimicrobial peptides. The vent mussel *B. azoricus *has been the subject of previous studies, in our laboratory, aiming at the characterization of its cellular and humoral immune reactions as a means to better understand physiological adaptations to vent environments. Given the limited immune gene expression information available for this non-conventional model organism, a query-based strategy was used to identify coding sequences within the DeepSeaVent database, for the selection and retrieval of nucleic acid information of cDNAs corresponding to genes potentially involved in immune and inflammatory reactions. Therefore, the selection of candidate genes presented in Table 2 was based on queries using specific descriptors matching immunity and inflammation-related criteria allowing thus the identification and retrieval of relevant cDNA sequences from the transcriptome library.

**Table 2:** *B. azoricus *genes putatively involved in immune response and inflammatory reactions.

Function	Gene Ontology n°	Gene Ontology description
**Recognition**		
Peptidoglycan Recognition protein (PGRP)	GO: 0008745	N-acetylmuramoyl-L-alanine amidase activity
Chitin binding protein	GO:0008061; GO:0006030	Chitin binding; chitin metabolic process
Galectin 4-like protein	GO:0005529	Sugar binding
Rhamnose-binding lectin	GO:0005529	Sugar binding
Thrombospondin-like glycoprotein	GO:0007155; GO:0033627	Cell adhesion; cell adhesion mediated by integrin
Glycoside hydrolase, Chitinase-like	GO:0005975	Carbohydrate metabolic process
Mannose-6-phosphate receptor	GO:0005537	Mannose binding
Contactin associated protein 2	GO:0007155; GO:0005515	Cell adhesion; protein binding
Tissue inhibitor of metalloproteinase	GO:0008191; GO:0005578	Metalloendopeptidase inhibitor activity; proteinaceous extracellular matrix
Serpin (serine protease inhibitor)	GO:0004867	Serine-type endopeptidase inhibitor activity
α_2_-Macroblobulin (thioester-containing protein)	GO:0004866	Endopeptidase inhibitor activity
Syndecan binding protein	GO:0007265; GO:0005137	Ras protein signal transduction; interleukin-5 receptor binding
Fibrinogen (pattern recognition receptor)	GO:0007165; GO:0005102	signal transduction; receptor binding
Ficolin (opsonin, contain fibrinogen and collagen-like domains)	GO:0007165;GO:0005102 GO:0008228	signal transduction; receptor binding; opsonization
Scavenger receptor cysteine-rich protein (SRCR)	GO:0005044	Scavenger receptor activity
LBP/BPI (LPS binding, Crassostrea homologue)	GO:0008289	Lipid binding
		
**Signaling**		
Toll-interleukin receptor	GO:0045087; GO:0007165	Innate immune response; signal transduction
Myd88	GO:0004888	Transmembrane receptor activity
TRAF (TNF receptor-associated factor)	GO:0007165; GO:0042981	Signal transduction; regulation of apoptosis
IRAK	GO:0019221; GO:0051092	Cytokine-mediated signaling pathway; regulation of NF-κB
MAPK	GO:0004672; GO:0006468	Protein kinase activity; protein amino acid phosphorylation
p38	GO:0004672; GO:0051403	Protein kinase activity; stress-activated MAPK cascade
Notch homologue	GO:0007411; GO: 0007219	Axon guidance; Notch signaling pathway
EGF receptor	GO:0007173; GO:0007165	Epidermal growth factor receptor signaling Pathway; signal transduction
TNF receptor	GO:0007165; GO:0042981	Signal transduction; regulation of apoptosis
Fibropellin homologue	GO:0005509; GO:0005515	Calcium ion binding; protein binding
Laminin_EGF	GO:0005539	Glycosaminoglycan binding
Cadherin (EGF domain containing)	GO:0016020; GO:0007156	Membrane;homophilic cell adhesion
Integrin (fibronectin receptor)	GO:0007155; GO:0007229	Cell adhesion; integrin mediated signaling pathway
		
**Transcription**		
Nuclear Factor κB inhibitor	IPR015681 (InterPro)	Regulation of NF-κB activity
STAT	GO:0004871; GO:0045449	Signal transducer activity; regulation of transcription
SH2 motif(Src homology 2	GO:0007165; GO:0018108	Signal transduction; peptidyl-tyrosine phosphorylation
P53	GO:0006915; GO:0034984	Apoptosis; cellular response to DNA damage stimulus
AP-1 (Proto-oncogene c-jun)	GO:0003700; GO:0045449	Transcription factor activity; regulation of transcription
Tal (Crassostrea homologue)	GO:0045449; GO:0030528	Regulation of transcription; transcription regulator activity
		
**Effector and modulator molecules**		
Defensin (big defensin)	GO:0006952	Defense response
Cytolysin	GO:0009404	Toxin metabolic process
Apolipoprotein (plasminogen)	GO:0007596; GO:0004252	Blood coagulation; serine-type endopeptidase activity
TNF (LPS-induced, α factor)	GO:0006955; GO:0006952	Immune response; defense response
Interferon	GO:0042742	Defense response to bacterium; regulation of innate immune response
TGF	GO:0006954; GO:0006917	Inflammatory response; induction of apoptosis
Glutatione peroxidase	GO:0004602; GO:0006979	Glutathione peroxidase activity; response to oxidative stress
Prostaglandin synthase/cyclooxygenase	GO:0006979; GO:0004601	Response to oxidative stress; peroxidase activity
Fibronectin	GO:0001968	Fibronectin binding
Metalloproteinase	GO:0004222; GO:0005578	Metalloendopeptidase activity; proteinaceous extracellular matrix
Metallothionein	GO:0046872	Metal ion binding
Ferritin	GO:0006879	Cellular iron ion homeostasis
Tenascin	GO:0009611; GO:0007155	Cell adhesion; response to wounding
Glucose-regulated protein 94	GO:0006950	Response to stress

### The identification of putative genes was based on GO annotation and querying the DeepSeaVent database

**Figure 3:**
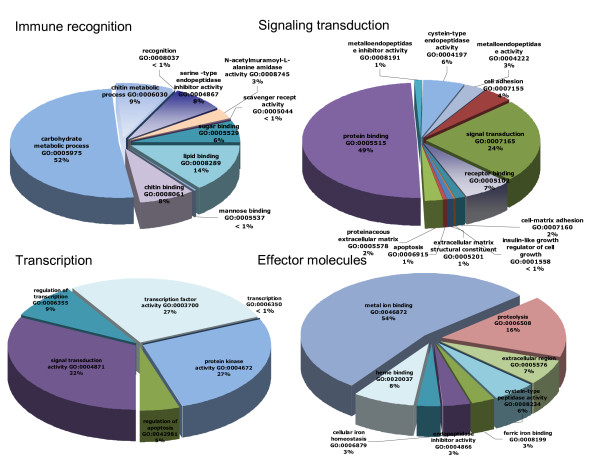
**Categorization of putative immune genes**. A proposed categorization of immune genes is illustrated, according to Gene Ontology terminology, into four functional classes of innate immunity constituents from *B. azoricus*: immune recognition, signal transduction, transcription and effector molecules.

A number of putative genes which have not previously been identified in *B. azoricus *were classified within the context of GO representation and to one of the following immune categories: immune recognition, signal transduction, transcription and effector molecules (Fig 3), providing thus a classification resource for the investigation of specific processes, functions or cellular structures involved in animal physiological responses, particularly immune and stress-related responses, as demonstrated for the *de novo *analysis of the *Acropora millepora *larval transcriptome [19].

**Figure 4:**
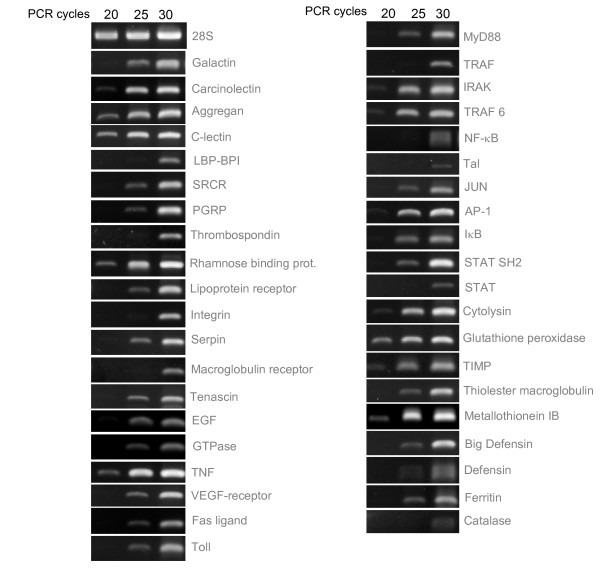
**Semi-quantitative Reverse Transcription-PCR (RT-PCR) of candidate genes**. Normalized cDNA obtained from reverse transcription of mRNA was used as template for PCR amplifications. Aliquots were taken from PCR reactions at 20, 25 and 30 cycles and analyzed by agarose gel electrophoresis.

**Figure 5:**
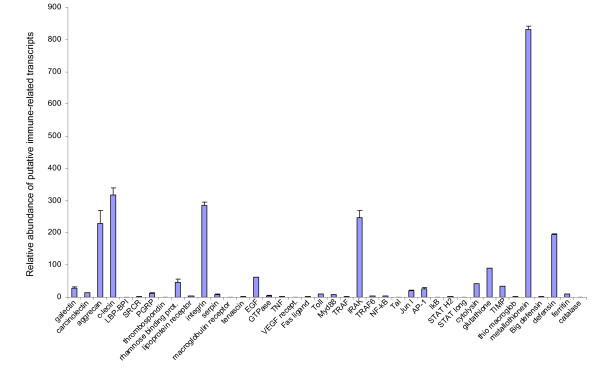
**Quantitative expression of putative immune and stress-related genes**. The quantitative expression of putative genes from vent mussel gills tissues was assessed by qPCR. Data were transferred to Excel files and plotted as histograms of fold expression of putative genes from non-normalized cDNA library. Results are mean ± SD (N = 3). Vertical bars represent the relative expression levels of putative transcripts using the 28S as control and normalization gene.

Subsequently, the physical counterpart of these putative genes was confirmed by semi-quantitative Reverse Transcription-PCR, using primers specifically designed on the basis of the newly identified EST sequences and using the same source of cDNA as for 454 pyrosequencing. Consequently, 454 sequencing artefacts could be discarded on the assumption that PCR amplifications of partial cDNA of the candidate genes were successfully obtained. Even though the transcriptome library was normalized, it is still qualitatively possible to visualize different cDNA abundance as PCR amplifications revealed different amplicon intensities for some of the confirmed genes, presumably due to different mRNAs prevalence in the normalized cDNA library (Fig 4). This was further analyzed by quantitative PCR (qPCR) utilizing non-normalized cDNA as template for amplification of immune-related genes. Indeed, results demonstrated the differential abundance of transcripts in the non-normalized transcriptome library confirming that genes tested in our study are not expressed at the same level and are likely representing the transcriptional activity at the time animals were collected from the bottom of the sea (Fig 5). Particularly, the metal-binding metallothionein gene was highly expressed in the non-normalized transcriptome library, probably reflecting the physiological need for continuous production of this protein to overcome the metal stress occurring at vent sites and to regulate intracellular levels of metals. Other immune response representatives also showed increased levels of expression as compared to the remaining genes studied. These include immune recognition molecule aggrecan and c-lectin, cell signalling molecule integrin, signal transduction complex component IRAK, and antibacterial protein defensin, (Fig 5), all providing evidence of a functional immune system in *B. azoricus*.

The selected putative genes are being currently investigated under controlled conditions in our laboratory, to assess the effect of long-term acclimatization in aquaria at atmospheric pressure, the effect of *de novo *hyperbaric stimulations in the IPOCAMP chamber and the effect of exposure to marine *Vibrio *bacteria, on *B. azoricus *transcript profiling experiments [20,21].

Furthermore, this search-based analysis was also particularly important to determine a "bacterial fingerprint" in *B. azoricus *gill tissues, since we expected the vent mussel to have a rich microorganismal community and more specifically a substantial accumulation of endosymbiont bacteria within its gill tissues. The selection of poly-A RNA as the starting material for our transcriptome library likely eliminated many potential microbial sequences. However, 3,522 contigs in DeepSeaVent presented protein match hits to bacterial phylotypes, supporting the evidence for the presence of bacteria in gill tissues of *B. azoricus*, and representing thus a potential bacterial fingerprint, most likely of chemoautotrophic nature, in deep-sea hydrothermal vent mussels. Additionally, a number of bacterial sequences were ascribed to several non-cultured marine bacteria, to chemolithoautotrophic, sulfur- or methane-oxidizing bacteria as evidenced, for instance, by the presence of the SoxB, SoxY, SoxH, methane monooxygenase, Biopolymer transport protein ExbD/TolR genes when querying our database using InterPro or GO terms as "methane" or "sulfur". Similarly, searches using the genera *Calyptogena *and *Riftia *names returned several hundreds of putative protein sequences, the majority of which associated to endosymbionts from the giant hydrothermal vesicomyid clam and vestimentiferan tubeworm, respectively from the East Pacific Rise hydrothermal region.

Such an approach has the potential to reveal sequences that have apparent bacterial origin including many genera of species that have been associated with mussel pathogens or as normal flora in the gut system [22]. In this case, mutualistic interaction between symbiont bacteria and their animal hosts may be taken to another level of analysis based on transcriptome sequencing. New genes involved in host recognition of endosymbionts and immune-effector mechanisms underlying host defense responses may shed light into understanding mutualism better and provide explanations as to how endosymbiont bacteria, living inside the bacteriocytes of vent mussels, are able to evade immune recognition, during early stages of acquisition and how mutualism is maintained. One possible explanation might lie within the immune response itself, where constitutive immune responses of bacteriocytes towards the dense population of endosymbionts, might be expressed at different levels of the rest of the body and therefore, keeping endosymbionts under control [22]. This hypothesis is currently under experimental consideration in our laboratory.

### Deep-sea vent mussel comparison to shallow water mussel

The DeepSeaVent database was compared to Mytibase, a recently created EST database from the shallow water mussel, *Mytilus galloprovincialis *[23]. The comparison was restricted to the amino-acid sequences with InterPro annotation from both databases and by setting the BLAST parameters to an E-value of 10^-5 ^and bit scores ranging from 90 to 200. Under such conditions 5,261 and 1,923 amino-acid sequences, respectively, were identified. Furthermore, the shared amino-acid sequences were analyzed in relation to the distribution of GO functional annotation categories, using the 4,120 sequences retrieved with a bit score of 120. The highest percentage of common GO-annotated sequences (50% sharing and above) between the two databases corresponded to the categories, Cell Killing, Cellular Component Biogenesis, Macromolecular Complex and Structural Molecule Activity (Table 3).

**Table 3:** Comparison between Mytibase and DeepSeaVent database

DeepSeaVent and Mytibase comparison				
E-value	Bit-score	Matched proteins with InterPro annotation		
10^-5^	90	5,261		
10^-5^	120	4,120		
10^-5^	200	1,923		

**Biological Process** **GO:0008150**		**DeepSeaVent**	**DeepSeaVent** **vs** **Mytibase**	**Shared percentage**

Cell killing		12	6	50.0
Immune system process		56	3	5.4
Death		14	2	14.3
Multicellular organismal process		39	6	15.4
Cellular component biogenesis		318	175	55.0
Cell wall organization or biogenesis		9	1	11.1

**Cellular Component** **GO:0005575**				

Virion		63	1	1.6
Macromolecular complex		1495	798	53.4
Virion part		63	1	1.6

**Molecular Function** **GO:0003674**				

Structural molecule activity		942	533	56.6
Transporter activity		570	101	17.7
Electron carrier activity		350	54	15.4
Enzyme regulator activity		161	22	13.7
Molecular transducer activity		110	6	5.5

The fixed E-value 10^-5 ^was kept for three different bit-scores (90,120 and 200). The number of contigs with GO ontology assignments shared between both databases is shown together with respective shared percentage

The biological significance of these findings was not immediately evaluated due the comparatively low amount of amino-acid sequences available in the Mytibase as compared to DeepSeaVent. However, representatives of broad GO categories are present in both Mytibase and DeepSeaVent databases, suggesting that mussels originated from distinct marine habitat may share common biological processes, cellular components and molecular functions.

Such inter-database computational analyses offers now the potential to unravel genes specifically involved in hydrostatic pressure and chemosynthetic environmental adaptations by comparing transcriptome profiles from two closely related Mytilid family members living in very distinct marine habitats.

## Conclusions

Comparison of our results with recently published transcriptomic studies on *B. azoricus *confirms the efficacy of 454 sequencing to reveal a large number of putative transcripts and significantly improve the genomic knowledge on this deep sea animal. The use of 454 pyrosequencing to develop a new EST collection containing potentially 39,425 new transcripts provides a new resource for genome-wide association studies of vent mussel physiological variations, which is the focus of ongoing projects in our laboratory, addressing in particular the molecular adaptation mechanisms of *B. azoricus *to deep-sea hydrothermal vent environments. This new resource now gathered in the DeepSeaVent database will set the stage for innovative work and the establishment of large scale expression studies to validate the deep-sea vent mussel as a *bone fide *experimental model to study the biology of adaptation to deep-sea hydrothermal vent environments.

## Methods

### Animals sampling

Mussels were collected from the hydrothermal vent field Lucky Strike (37° 13.52' N, 32° 26.18' W; 1700 m depth), on the Mid-Atlantic Ridge (MAR), with the American R/V *Revelle *using the ROV Jason II (Woods Hole Oceanographic Institution), during the MAR08 cruise (July 9^th ^- August 16^th ^2008) led by Chief Scientist Dr Anna-Louise Reysenbach. Once the mussels were brought to the surface, they were immediately processed onboard for subsequent manipulation of RNA or immediately stored at -80°C for long-term preservation of tissue samples.

### cDNA library construction and pyrosequencing

Gill tissues from 6 different animals were dissected from -80°C preserved animals and processed for total RNA extraction using the RiboPure(tm) kit (Ambion^(r)^, Austin, TX). The quality of total RNA was verified on a 1.4% (w/v) agarose-MOPS-formaldehyde denaturating gels and by assessing the A_260/280 _and A_260/230 _ratios using the NanoVue spectrophotometer (GE Healthcare, Piscataway, NJ). Poly-A RNA was extracted from each total RNA sample using the Poly(A)Purist(tm) mRNA Purification Kit according to manufacturer's instructions (Ambion Inc, Applied Biosystems). The mRNA from each gill tissue sample from all 6 animals (approximately 20-50 μg/ml) was pooled and used as the source of starting material for cDNA synthesis and the production of normalized cDNA intended for 454 sequencing. The normalization process was performed by Evrogen (Moscow, Russia) and based on the SMART double-stranded cDNA synthesis methodology using a modified template-switching approach that allows the introduction of known adapter sequences to both ends of the first-strand cDNA. cDNA was further amplified by PCR and normalized using Duplex-Specific Nuclease-technology [24,25].

Four micrograms of normalized cDNA were sequenced in a full plate of 454 GS FLX Titanium according to the standard manufacturer's instructions (Roche-454 Life Sciences, Brandford, CT, USA) at Biocant (Cantanhede, Portugal).

### Sequence processing, data analysis and functional annotation

Following 454 sequencing, the quality trimming and size selection of reads were determined by the 454 software after which the SMART adaptor sequences were removed from reads using a custom script and the poly-A masked using MIRA, to assure correct assembly of raw sequencing reads [26]. A total of 778,996 quality reads were subjected to the MIRA assembler [26] (version 3.0.5), with default parameters, yet only 582,650 reads were assembled. For some reads, after masking the poly-A, the sequence length was shorter than 40 bp, otherwise the minimum length assumed by the MIRA default parameter settings. The software also disregards all reads that do not match any other read or that belong to the megahub group, i.e. a read that is massively repetitive with respect to other reads. Such reads are considered singlets and were not included in the final assembly result. On the other hand, singletons were defined as reads that match other reads but neither do have sufficient coverage nor present conditions to assemble with them. The entire set of reads used for final assembly was submitted to the NCBI Sequence Read Archive under the accession n° SRA024338 (Submission: SRA024338.1/Bathymodiolus azoricus).

**Figure 6:**
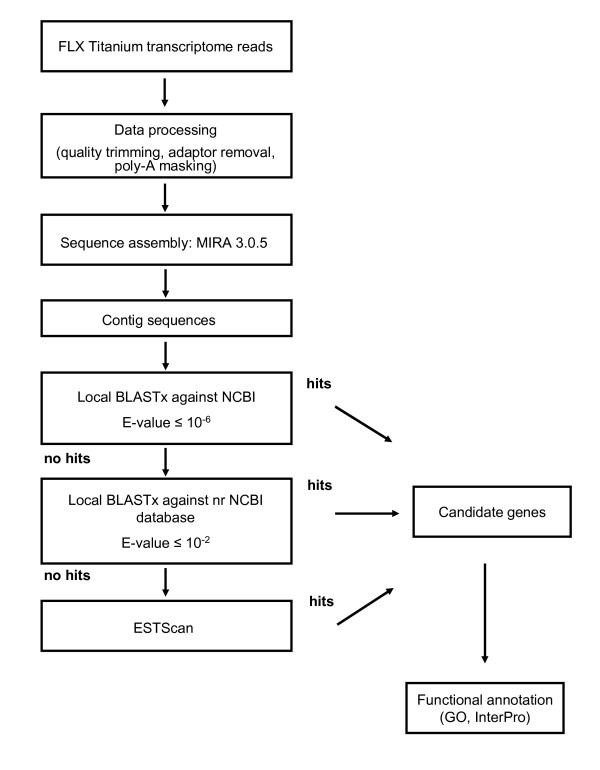
**Flowgram representing data processing pipeline for *de novo *transcriptome assembly and annotation of *B. azoricus *gill tissues**.

The translation frame of the contigs was determined through queries against the NCBI non redundant protein database using BLASTx with an E-value of 10^-6 ^and assessing the best twenty five hits. Contigs without hits were submitted again to BLASTx homology searches against the NCBI nr database with a higher E-value cut-off set at 10^-2^. Sequences with a translation frame identification derived from the two previous searches were used to establish the preferential codon usage in *B. azoricus *based on which the software ESTScan detected further potential transcripts from the two previous sets of sequences with yet no BLASTx matches. This procedure originated a third set of sequences with putative amino-acid translation. To illustrate the outline flow for the transcriptome annotation procedure a diagram is shown in Fig 6.

The entire collection of sequences of at least 30 amino-acid long, resulting from the BLASTx and the ESTScan procedures, was processed by InterProScan for the prediction of protein domain signatures and Gene Ontology terms. All the results were compiled into a SQL database developed as an information management system.

The distribution of sequences into GO categories was calculated at each level and were passed to the parent GO at the top of the broad ontology domains, considering that each single assignment into a GO child was only counted once in the total sum. This information was also useful to establish the number of amino-acid sequences shared between the two Mytibase and DeepSeaVent databases.

**Figure 7:**
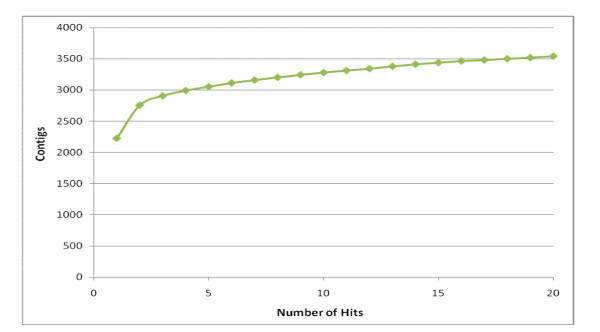
**Number of contigs presenting a bacterial match after BLASTx query**.

The retrieval of sequence information matching bacterial phylotypes was based on a convergent analysis of bacterial hits among the best BLASTx hits. The number of positive hits was calculated and plotted according to the position of best hit ranging from the first to the twentieth. A convergent number of bacterial hits was found at 3,522 contigs (Fig 7). The unique identifiers of the 3,522 contigs (gi accession number) were retrieved and translated into the taxon ID using the information provided by NCBI. A custom script based on the BioPerl module bio::db::taxonomy, version 1.6 [27], was used to link the taxon of interest to the superkingdom Bacteria and verify whether or not the BLASTx hit corresponded indeed to the canonical Eubacterial sequences.

### Identification of candidate genes putatively associated with immunity and inflammatory reactions

Candidate genes were selected following queries performed to our database using distinct search descriptors based on BLAST hit descriptions; GO descriptions; Interpro descriptions, GO and Interpro identification numbers. Queries were aimed at the identification of genes described in the literature as being related to immunity and inflammatory reactions. Reverse-Transcription followed by Polymerase Chain Reaction (RT-PCR) was performed to confirm the physical identity of the selected genes recognized among the 454-ESTs using the normalized cDNA. 2 μg of total RNA, initially used to generate the transcriptome library, were reverse transcribed using the ThermoScript™ RT-PCR system (Invitrogen, CA, USA) following manufacturer's instructions. All the contig sequences from putative immune genes were submitted to GenBank and assigned the accession numbers HM756110 to HM756130; HM756132 to HM756134; HM756136 to HM756149; HM756151 and HM756152 (Table 4).

**Table 4:** Forward and reverse primer sequences used in semi quantitative RT-PCR analyses.

Candidate gene	GenBank acc. no	5'-3' forward primer	5'-3' reverse primer
**RECOGNITION**			
Galectin	HM756110	CTCCGGCGGGAGGGAATCCA	AGTGGAAGCTGGGGTTCCGAGG
Carcinolectin	HM756111	CGGATACAGTGGCACGGCAG	TGATACCAACGAGCACCAGCAC
Aggrecan	HM756112	TGCAAGCGGATACCCGGTAAA	ATCAACGCAGAGTGGCCGAG
C-lectin	HM756113	AGGCTTGGGATAGGCACATGGA	ACGATTCACCCGAACAGAGTTGG
LBP/BPI	HM756114	GCTTCACTGATACTGCTTGCCC	CCACGGTGGAGCAGCATGGA
SRCR	HM756115	TGATTCGATACCAAGGACCCAAAGGT	TGTCAACTCCGGCTATTCCAGGT
PGRP	HM756116	TCACACGGAAGGAGGAGCGT	AGGGCTGCCTTGGATGGTGT
Thrombospondin	HM756117	TGCTGCGACCCATTTCTGTGA	GTGAGGAGTTCCACTGGTGAGGG
Rhamnose-binding lectin	HM756118	ACAATGGGTTGATTTGTTTGCCGA	CCGGGGGCCTGAAAGTTGGT
			
**SIGNALING**			
Lipoprotein receptor	HM756119	CAGAGCCATCCACTTTGGCGG	AGGTCTACACCTTCCAGCAGCA
Integrin	HM756120	ACGGCCGGGGAGAAGTTGAA	CGCAGTCACACGTTCCACAGAC
Serpin	HM756121	AGGGTTGTGCGTGAAGTGGA	TCTCAAAGCGAGGCTGCCAGA
α-macroglobulin receptor	HM756122	CATTACGGCCGGGGCAAAGG	TGCTGGCTCTCTCAGCTCGG
Tenascin	HM756123	CATTACGGCCGGGGGTTGTA	AGTCGGAACAGTCCTTTGGGT
EGF	HM756124	GGGACACATTGCGAAACGGC	TTGCCCCGTAAATCCAGGCA
GTPase	HM756125	ATTACGGCCGGGGGACACAC	TTCGGCATCCTGGCACTTCG
TNF factor	HM756126	GGGATTAGGCAACACCCAAGCC	CCGCCACAGTACAGCCAACC
VEGF receptor	HM756127	AGCTGCATGGAGACTTGAACCAGA	AGGTGGGGTGGTACTTGCTCC
Fas ligand	HM756128	CGATTCGCTAGGACCGGGGA	AGTCATTGGCGGTACTCCACACA
Toll	HM756129	AGGAGGACTCGGATGACACAGC	ACTCCGGAACTTGGAGAGCACG
MyD88	HM756130	CTGCCACACCCAACAACGCA	TCGAGACTGAGGTTCTCGCACA
TRAF	HM756132	CCCAACGACAGCCTTCTTTGACG	ATTACGGCCGGGGGCTTGTT
IRAK	HM756133	GAGTGGCCATTACGGCCGGG	GCTTGCATCGATCTGGCGGGT
TRAF 6	HM756134	CACCTATTTCCGCTTCCCGCC	TGGAGGGTGGTGGTGCTCTT
			
**TRANSCRIPTION**			
NF-κB	HM756136	CCAAATGATGCACCTGCTCTTTTCAGT	CATTACGGCCGGGGAAGGGA
Tal	HM756137	GTTGACGCCATCGCTCTCGG	GCCATTACGGCCGGGGTTTA
Jun	HM756138	CGCCAACACCGACACAGTTCA	AACCCCCGGGGAGTGTTGTT
AP-1	HM756139	TGCAGCTACACGGTTTCTGGC	TCGGCAACAACACTCCCC
I-κB	HM756140	TGAGGCAGCACTGAACGGAC	CGCAGAGTGTGCCAACAGCA
STAT long form	HM756141	ACGGCCGGGGTAAAGCTGAA	ACAAATCCAGCCACATGCCCA
STAT (SH2 motif)	HM756142	AGCGTCAAACACGACAGACGA	AGACCACGCCCTGTTTCAGC
			
**EFFECTOR**			
Cytolysin	HM756143	CGGTTGCTGTGTAGCCGCAT	GGCGTCCAGAGACCGGAGTT
Glutathione peroxidase	HM756144	TTAACGGCGTCGTCGCTTGG	TGGCTTCTCTCTGAGGAACAACTG
TIMP	HM756145	TGTCCCATGGGTCTGGAACGG	TCAGCCTGTTCCTCTTGGCATT
Thiolester- macroglobulin	HM756146	CTGGCTCTCTCAGCTCGGCA	GGGCACTCTCCGGTCTTGGT
Metallothionein 1B	HM756147	TCGGCACTGTCCACACAAAACC	CAACCGGAAGCGGATGTGGC
Big Defensin	HM756148	CCGGGGGCGATTGCCTTTC	ACCAAGGCCCAAAATGCAGC
Defensin	HM756149	AACGCAGAGTGGGCCATACG	TCACTGGTGCGAACCGTTTGT
Ferritin	HM756151	TCAACGCAGAGTGGGGCCAT	GCGGTTCAGAAGTTGTTGTCACG
Catalase	HM756152	CATGTTAGCAGGCACTCCAGACC	TACGGCCGGGGGAAAAAGGT

Based on sequences retrieved from the DeepSeaVent database, primers were designed to confirm, in RT-PCR experiments, the physical counterpart of *B. azoricus *genes putatively associated with immunity and inflammatory reactions.

Primers targeting the immune candidate genes were designed using the Primer-Blast [28] from NCBI http://www.ncbi.nlm.nih.gov/, specifying an expected PCR product of 200-300 bp and primer annealing temperatures between 56°C and 58°C. 25 μl PCR volume reactions were set with 1 μl of each forward and reverse primers (0.5 μM final concentration) and using a 2× PCR mix from PROMEGA (Madison, USA). PCR cycling conditions were according to Bettencourt et al. 2009 [29]. PCR products were examined by agarose gel electrophoresis according to standard methods.

Quantitative PCR (qPCR) was further used to assess and quantify the relative expression of the candidate genes previously tested on semi-quantitative RT-PCR. The non-normalized cDNA was obtained as previously described and consisting of the same cDNA utilized for subsequent normalization and 454 sequencing procedure. Quantitative PCR reactions were performed on the CFX96™ Real Time PCR System mounted onto the C1000 Thermal Cycling platform (Bio-Rad, CA, USA). Amplifications were carried out using 0.5 μl (10 μM) of the specific primers as for semi-quantitative PCR and mixed to 10 μl of SsoFast™ Eva Green SuperMix (SYBR based system, Bio-Rad) and 50 ng of cDNA in a final volume of 20 μl. PCR cycling conditions were 95°C for 3 min, followed by 35 cycles of 95 °C 10 s, 58 °C 15 s and 72 °C 30 s. 6 replicates were performed for each gene tested in real time PCR reactions. Melt curves profiles were analyzed for each gene tested. The 28S rRNA gene was used as the housekeeping gene and for normalization of expression of gene of interest or immune-related target genes. The comparative CT method (ΔΔC_T_) for the relative quantification of gene expression was used for assessing the normalized expression value of immune-related genes using the 28S rRNA as the control transcript (CFX Manager™ Software, Bio-Rad). Data were transferred to Excel files and plotted as histograms of normalized fold expression of target genes.

## Authors' contributions

RB conceived and designed the study, collected samples, participated in the data analysis and drafted the manuscript. MP developed the pipe-line analysis for all functional annotation and developed the SQL database. CE helped conceive the study and the manuscript drafting, oversaw sequencing reaction and participated in sequence analyses. PG participated in the preparation of cDNA for 454 sequencing reaction and set-up of the 454 equipment. MA participated in the gene confirmation experiments. TS provided logistics and details for sampling of *B. azoricus *and participated in the discussion of sequencing results and manuscript drafting. RS contributed to the general coordination of the study in the Azores and helped drafting the final manuscript. All authors read and approved the final manuscript.

## References

[B1] Childress JJ Fisher CR The biology of hydrothermal vent animals: physiology, biochemistry, and autotrophic symbioses Oceanogr Mar Biol Annu Rev 1992 30 337 441

[B2] Desbruyères D Biscoito M Caprais JC Colaço A Comtet T Crassous Ph Fouquet Y Khripounoff A Le Bris N Olu K Riso R Sarradin PM Segonzac M Vangriesheim A Variations in deep-sea hydrothermal vent communities on the Mid-Atlantic Ridge near the Azores plateau Deep Sea Res Pt I 2001 48 1325 1346 10.1016/S0967-0637(00)00083-2

[B3] Salerno JL Macko SA Hallam SJ Bright M Won YJ McKiness Z Van Dover CL Characterization of Symbiont Populations in Life-History Stages of Mussels From Chemosynthetic Environments Biol Bull 2005 208 145 155 10.2307/3593123 15837964

[B4] Won YJ Hallam SJ O'Mullan GD Pan IL Buck KR Vrijenhoek RC Environmental Acquisition of Thiotrophic Endosymbionts by Deep-Sea Mussels of the Genus *Bathymodiolus* Appl Environ Microbiol 2003 69 6785 6792 10.1128/AEM.69.11.6785-6792.2003 14602641 PMC262266

[B5] Duperron S Bergin C Zielinski F Blazejak A Pernthaler A McKiness ZP Dechaine E Cavanaugh CM Dubilier N A dual symbiosis shared by two mussel species, *Bathymodiolus azoricus *and *Bathymodiolus puteoserpentis *(Bivalvia: Mytilidae), from hydrothermal vents along the northern Mid-Atlantic Ridge Environ Microbiol 2006 8 1441 47 10.1111/j.1462-2920.2006.01038.x 16872406

[B6] Fiala-Medioni A McKiness ZP Dando P Boulegue J Mariotti A Alayse-Danet AM Robinson JJ Cavanaugh CM Ultrastructural, biochemical, and immunological characterization of two populations of the mytilid mussel *Bathymodiolus azoricus *from the Mid-Atlantic Ridge: evidence for a dual symbiosis Mar Biol 2002 141 1035 1043 10.1007/s00227-002-0903-9

[B7] Distel DL Lee HKW Cavanaugh CM Intracellular coexistence of methanotrophic and thioautotrophic bacteria in a hydrothermal vent mussel Proc Natl Acad Sci USA 1995 92 9598 9602 10.1073/pnas.92.21.9598 7568180 PMC40849

[B8] Bettencourt R Roch P Stefanni S Rosa D Colaço A Serrão R Santos Deep sea immunity: unveiling immune constituents from the hydrothermal vent mussel *Bathymodiolus azoricus* Mar Environ Res 2007 64 108 27 10.1016/j.marenvres.2006.12.010 17291578

[B9] Kadar E Powell J Post-capture investigation of the hydrothermal vent macro-invertebrates to study adaptation to extreme environments Rev Env Sci Biotechnol 2006 5 193 201 10.1007/s11157-006-0006-z

[B10] Tanguy A Bierne N Saavedra C Pina B Bachère E Kube M Bazin E Bonhomme F Boudry P Boulo V Boutet I Cancela L Dossat C Favrel P Huvet A Jarque S Jollivet D Klages S Lapègue S Leite R Moal J Moraga D Reinhardt R Samain J-F Zouros E Canario A Increasing genomic information in bivalves through new EST collections in four species: Development of new genetic markers for environmental studies and genome evolution Gene 2008 408 27 36 10.1016/j.gene.2007.10.021 18054177

[B11] Boutet I Jollivet D Shillito B Moraga D Tanguy A Molecular identification of differentially regulated genes in the hydrothermal-vent species *Bathymodiolus thermophilus *and *Paralvinella pandorae *in response to temperature BMC Genomics 2009 10 222 10.1186/1471-2164-10-222 19439073 PMC2689276

[B12] Altschul SF Gish W Miller W Myers EW Lipman DJ Basic local alignment search tool J Mol Biol 1990 215 403 10 2231712 10.1016/S0022-2836(05)80360-2

[B13] Iseli C Jongeneel CV Bucher P ESTScan: a program for detecting, evaluating, and reconstructing potential coding regions in EST sequences Proc Int Conf Intell Syst Mol Biol 1999 138 48 10786296

[B14] Apweiler R Biswas M Fleischmann W Kanapin A Karavidopoulou Y Kersey P Kriventseva EV Mittard V Mulder N Phan I Zdobnov E Proteome Analysis Database: online application of InterPro and CluSTr for the functional classification of proteins in whole genomes Nucleic Acids Res 2001 29 44 48 10.1093/nar/29.1.44 11125045 PMC29822

[B15] Hunter S Apweiler R Attwood TK Bairoch A Bateman A Binns D Bork P Das U Daugherty L Duquenne L Finn RD Gough J Haft D Hulo N Kahn D Kelly E Laugraud A Letunic I Lonsdale D Lopez R Madera M Maslen J McAnulla C McDowall J Mistry J Mitchell A Mulder N Natale D Orengo C Quinn AF Selengut JD Sigrist CJA Thimma M Thomas PD Valentin F Wilson D Wu CH Yeats C InterPro: the integrative protein signature database Nucleic Acids Res 2008 37 211 215 10.1093/nar/gkn785 PMC2686546 18940856

[B16] Ashburner M Ball CA Blake JA Botstein D Butler H Cherry JM Davis AP Dolinski K Dwight SS Eppig JT Harris MA Hill DP Issel-Tarver L Kasarskis A Lewis S Matese JC Richardson JE Ringwald M Rubin GM Sherlock G Gene Ontology: tool for the unification of biology Nature Genet 2000 25 25 29 10.1038/75556 10802651 PMC3037419

[B17] Rast JP Smith LC Loza-Coll M Hibino T Litman GW Genomic insights into the immune system of the sea urchin Science 2006 10 952 956 10.1126/science.1134301 PMC3707132 17095692

[B18] Iwanaga S Lee BL Recent Advances in the Innate Immunity of Invertebrate Animals J Biochem Mol Bio 2005 38 128 150 10.5483/bmbrep.2005.38.2.128 15826490

[B19] Meyer E Aglyamova GV Wang S Buchanan-Carter J Abrego D Colbourne JK Willis BL Matz MV Sequencing and de novo analysis of a coral larval transcriptome using 454 GSFlx BMC Genomics 2009 10 219 10.1186/1471-2164-10-219 19435504 PMC2689275

[B20] Kadar E Tschuschke IG Checa A Post-capture hyperbaric stimulations to study the mechanism of shell regeneration of the deep-sea hydrothermal vent mussel *Bathymodiolus azoricus *(Bivalvia: Mytilidae) J Exp Mar Biol Ecol 2008 364 80 90 10.1016/j.jembe.2008.07.028

[B21] Bettencourt R Costa V Laranjo M Rosa D Pires L Colaço A Lopes H Serrão R Santos Out of the deep sea into a land-based aquarium environment: investigating physiological adaptations in the hydrothermal vent mussel *Bathymodiolus azoricus* ICES J Mar Sci 2010 *(first published online August 16, 2010)*

[B22] Stuart R Rolff J Immune function keeps endosymbionts under control J Biol 2008 7 28 10.1186/jbiol88 18947377 PMC2776399

[B23] Venier P De Pitta C Bernante F Varotto L De Nardi B Bovo Roch G Novoa B Figueras A Pallavicini A Lanfranchi G MytiBase: a knowledgebase of mussel (*M. galloprovincialis*) transcribed sequences BMC genomics 2009 10 72 10.1186/1471-2164-10-72 19203376 PMC2657158

[B24] Shcheglov A Zhulidov P Bogdanova E Shagin D Buzdin A, Lukyanov S Normalization of cDNA Libraries Nucleic Acids Hybridization: Modern Applications 2007 Dordrecht, Springer 97 124 full_text

[B25] Zhulidov PA Bogdanova EA Shcheglov AS Shagina IA Wagner LL Khazpekov GL Kozhemyako VV Lukyanov SA Shagin DA A method for the preparation of normalized cDNA libraries enriched with full-length sequences Bioorg Khim 2005 31 186 94 15889793 10.1007/s11171-005-0023-7

[B26] Chevreux B Pfisterer T Drescher B Driesel AJ Müller WE Wetter T Suhai S Using the miraEST Assembler for Reliable and Automated mRNA Transcript Assembly and SNP Detection in Sequenced ESTs Genome Res 2004 14 1147 59 10.1101/gr.1917404 15140833 PMC419793

[B27] Stajich JE Block D Boulez K Brenner SE Chervitz SA Dagdigian C Fuellen G Gilbert JG Korf I Lapp H Lehväslaiho H Matsalla C Mungall CJ Osborne BI Pocock MR Schattner P Senger M Stein LD Stupka E Wilkinson MD Birney E The Bioperl toolkit: Perl modules for the life sciences Genome Res 2002 12 1611 1618 10.1101/gr.361602 12368254 PMC187536

[B28] Rozen S Skaletsky H Primer3 on the WWW for general users and for biologist programmers Methods Mol Biol 2000 132 365 86 10547847 10.1385/1-59259-192-2:365

[B29] Bettencourt R Dando P Collins P Costa V Allam B Serrão R Santos Innate Immunity in the deep sea hydrothermal vent mussel *Bathymodiolus azoricus* Comp Biochem Physiol A 2009 152 278 89 10.1016/j.cbpa.2008.10.022 19041413

